# Comparison of feasibility, inter-instrument agreement, and prognostic value of four frailty assessment instruments in geriatric colorectal cancer patients: a 3-year multicenter prospective cohort study

**DOI:** 10.3389/fonc.2026.1878424

**Published:** 2026-10-09

**Authors:** Lei Ma, Feiyu Shi, Chenhao Hu, Kexue Wang, Lei Zhang, Penghong Qu, Junjun She

**Affiliations:** 1 Department of General Surgery, The First Affiliated Hospital of Xi'an Jiao Tong University, Xi'an, China; 2 Department of Anesthesiology and Perioperative Medicine, The First Affiliated Hospital of Xi'an Jiao Tong University, Xi'an, China

**Keywords:** adverse outcomes, cohort study, colorectal cancer, frailty, geriatric patients

## Abstract

**Background:**

Although multiple instruments exist to assess frailty, it remains unclear whether the association between frailty and clinical outcomes in older colorectal cancer (CRC) patients varies by the assessment tool used. Accordingly, the aim of this study was to compare the feasibility and clinical utility of four common frailty scales in this patient population.

**Methods:**

We conducted a 3-year prospective cohort study involving geriatric patients (≥65 years) with CRC. Preoperative frailty was assessed using four established scales: the Clinical Frailty Scale (CFS), the Frailty Phenotype (FP), the Geriatric-8 (G-8) scale, and the 32-item Frailty Index (FI). The differences in the perioperative outcomes and postoperative one-year all-cause mortality rate were compared across the four scoring systems.

**Results:**

A total of 1,096 patients were included in the primary analysis. The effective response rates and median completion times for the four frailty scales were as follows: CFS (91.1%; 6 min), FP (65.7%; 10 min), G-8 (71.6%; 8 min), and 32-item FI (58.3%; 18 min). The final analysis included 615 patients who completed all assessments. Frailty prevalence was estimated at 27.0% (CFS), 23.6% (FP), 33.0% (G-8), and 36.9% (FI). The four frailty scales demonstrated good predictive efficacy for postoperative clinically relevant complications, postoperative recovery, 30-day readmission, and one-year all-cause mortality. Multivariate regression analysis confirmed that the G-8 scale (OR = 2.412, 95%CI: 1.300–4.474, *P* = 0.008) and 32-item FI (OR = 3.035, 95%CI: 1.359–6.779, *P* = 0.017) served as independent risk factors for postoperative clinically relevant complications. Only the 32-item FI emerged as an independent predictor of 1-year all-cause mortality (HR = 2.777, 95%CI: 1.247–6.218, *P* = 0.020).

**Conclusion:**

In conclusion, all four frailty scales demonstrated robust discriminatory capacity for clinical outcomes in older CRC patients. Although the 32-item FI exhibited a stronger independent correlation with adverse outcomes following multivariate adjustment, its clinical utility must be weighed against a longer administration time. This study provides crucial evidence to guide the selection of an appropriate frailty assessment tool based on specific clinical contexts.

## Introduction

The world is undergoing a significant demographic transition, marked by increasing life expectancy and a growing aging population. In 2020, the population aged 65 years or older stood at 727 million and is projected to rise to 1.5 billion by 2050 (1). As a common digestive tract tumor in older adults, colorectal cancer (CRC) ranks third in global incidence and second in mortality among all cancers (2, 3). The aging process involves progressive physiological decline, characterized by reduced immune function, diminished organ reserve capacity, and decreased physical performance, collectively impairing the response to metabolic stress (4). These alterations at the molecular, cellular, and organ levels, combined with the metabolic demands imposed by the tumor itself, impair tolerance to standard cancer therapies. Previous studies have confirmed that older patients with CRC experience higher rates of adverse clinical events than younger patients, including increased postoperative complications, prolonged recovery, more frequent chemotherapy side effects, and poorer overall prognosis (5–7). Therefore, optimizing patient stratification to enable personalized therapeutic strategies represents a key challenge in managing this growing population.

In fact, the American Society of Clinical Oncology (8) and the International Society of Geriatric Oncology (9) routinely recommend a geriatric assessment (GA) for patients aged 65 years or older prior to treatment to inform clinical decision-making. Frailty, a core component of GA, is characterized as a multidimensional syndrome of diminished stress resilience resulting from multisystem decline and loss of homeostatic balance. The utility of frailty for risk stratification and predicting adverse events in surgical cancer patients has been demonstrated in previous studies, with documented associations to postoperative complications (10), adverse drug reactions (11), and mortality (12). Importantly, emerging evidence suggests that frailty may be reversible through early identification and targeted interventions (13). Supporting this, an international multicenter randomized controlled trial demonstrated that prehabilitation programs based on risk stratification can ameliorate preoperative frailty in CRC patients, ultimately reducing postoperative complications and accelerating recovery (14). A standardized frailty assessment embedded in evidence-based pre-rehabilitation programs within geriatric surgical pathways thus represents a promising strategy to improve outcomes in this vulnerable population. Consequently, the selection of an appropriate frailty assessment tool is critical for optimizing the care of older CRC patients.

Although a wide array of frailty assessment instruments has been developed, no universal gold standard exists for preoperative risk stratification among elderly patients with CRC (15). Clinicians continue to face controversy when selecting optimal frailty screening tools, attributable to substantial heterogeneity across existing scales—including divergent target populations, intended clinical scenarios, and core evaluation endpoints. Such inconsistencies impede the standardized incorporation of frailty evaluation into routine perioperative workflows. Against this backdrop, head-to-head comparisons examining the practical feasibility and prognostic performance of different frailty tools within older CRC cohorts are urgently needed.

International frailty research is predominantly built upon two well-recognized conceptual frameworks: the physical phenotype model, exemplified by the Fried Phenotype (FP), and the cumulative deficit model, represented by the Frailty Index (FI) (16). Beyond these two classic systems, the Geriatric-8 (G-8) scale was purpose-built for rapid frailty screening in older adults with malignancies (17), whereas the Clinical Frailty Scale (CFS) is widely favored for its brevity and bedside practicality (18). Given their fundamentally distinct theoretical underpinnings and complementary clinical positioning, we selected these four representative tools for comprehensive comparative analysis in the present study. To the best of our knowledge, no prior investigation has simultaneously benchmarked the discriminative capacity of this panel of frailty instruments for predicting adverse clinical outcomes in older CRC populations.

Accordingly, we therefore designed this prospective cohort study to head-to-head compare four widely adopted frailty assessment tools: CFS, FP, G-8, and 32-item FI. We aimed to evaluate their practical feasibility, clinical applicability, and discriminative power for predicting postoperative recovery and short-term prognostic events among older patients with CRC.

## Methods

### Study design and patients

This prospective multicenter cohort study was conducted at the First Affiliated Hospital of Xi'an Jiaotong University and its affiliated Yulin Hospital between June 2022 and December 2024. Patients aged 65 years or older with a diagnosis of CRC, who were able to understand and cooperate with the study procedures, were included. Exclusion criteria were as follows (1): no surgical intervention (2), local excision only (3), palliative surgery (4), incomplete clinicopathological or follow-up data, or (5) incomplete questionnaire submission or invalid response patterns. The patient screening process is illustrated in **Figure 1**. Written informed consent was obtained from all participants, and the study was approved by the Ethics Committee of the First Affiliated Hospital of Xi'an Jiaotong University (No. XJTU1AF2020LSL-004). This study was conducted in accordance with the principles of the 2013 revision of the Declaration of Helsinki and the Declaration of Istanbul.

**Figure 1:**
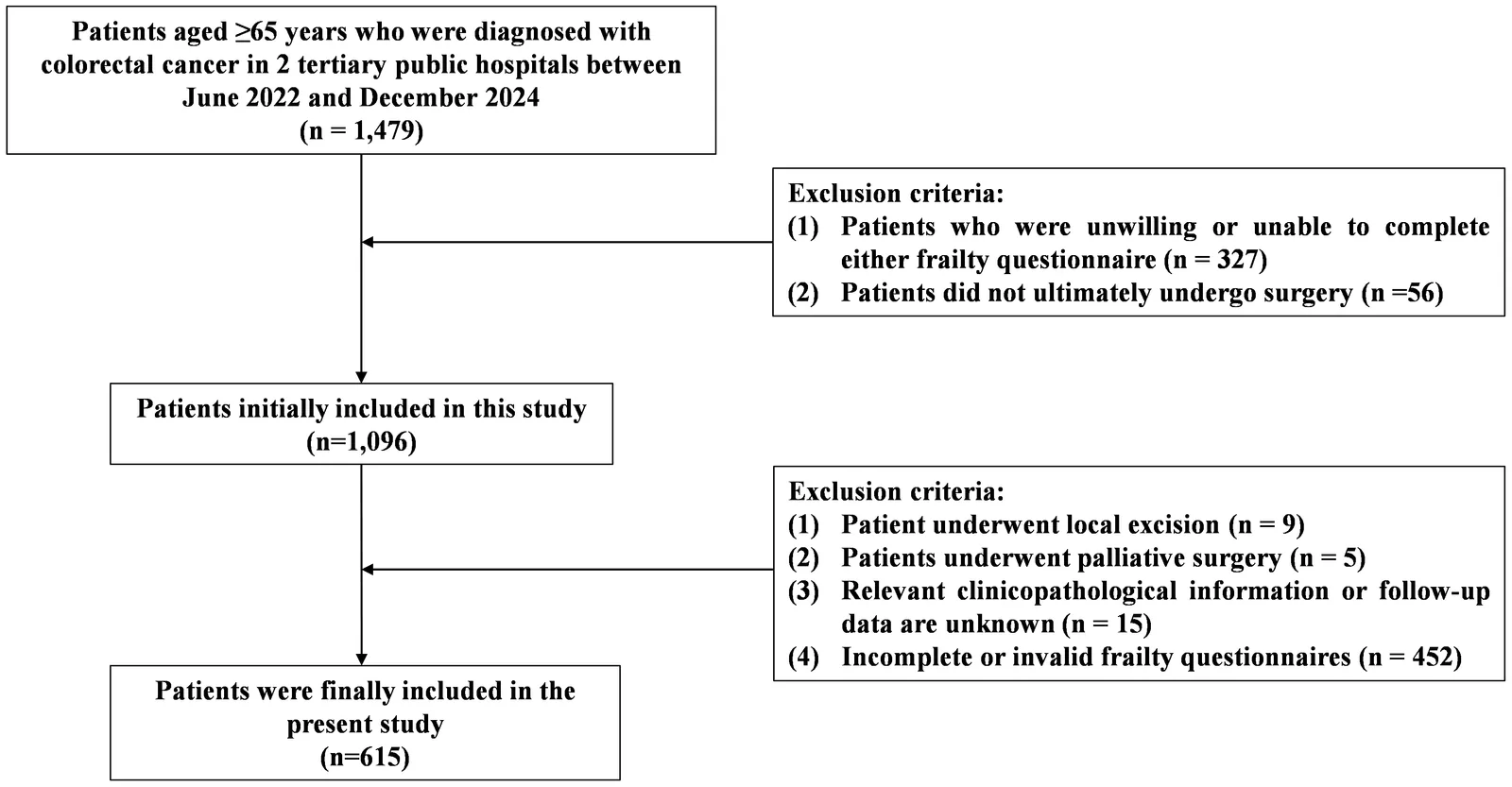
The inclusion criteria flowchart of study patients.

### Frailty assessment tools

The CFS, developed by a Canadian research institution, is a 9-point global assessment tool (18). It categorizes individuals based on a comprehensive evaluation of their disease severity, energy level, physical activity, and functional status. The scale, which ranges from 1 (very healthy) to 9 (terminally ill), indicates a higher degree of frailty with increasing scores. For this study, scores of 1–2 were defined as “fit," 3–4 as “pre-frail,” and 5 or higher as “frail”.

The FP, proposed by Fried in 2001, is a composite assessment instrument based on the frailty phenotype model (19). It evaluates five key components: unintentional weight loss, self-reported exhaustion, grip strength, walking speed, and low physical activity. One point is assigned for each criterion present. The total score ranges from 0 to 5, with scores of 0 classified as “fit," 1–2 as “pre-frail,” and ≥ 3 as “frail”.

The G-8 scale comprises eight items: age and seven clinical elements from the Mini-Nutritional Assessment—specifically, food intake, weight loss, mobility, neuropsychological comorbidities, body mass index, drug prescriptions, and self-reported health status (17). The total score can range from 0 to 17, thereby denoting greater frailty severity with decreasing scores. In this study, we adopted the three-category classification scheme from prior literature: scores >14 were categorized as fit, 11–14 as pre-frail, and <11 as frail (20, 21).

The present study employed a 32-item FI, which has been validated in prior research (22, 23). This index encompasses a range of health domains, including underlying diseases, medication use, psychological and cognitive status, physical function, and activities of daily living. The total score quantifies the ratio of existing deficits relative to the total number of deficits (32 items) assessed for each participant, with the score ranging from 0.0 (no deficits present) to 1 (all deficits present). Consistent with previous literature (24, 25), patients were stratified into three frailty states using the following FI cut-offs: < 0.10 for 'fit', 0.10 to < 0.25 for 'pre-frail', and ≥ 0.25 for 'frail'.

As the CFS contains only a single item, internal consistency analysis and common method bias testing could not be performed for this scale. For the other three multi-item frailty tools, the explanatory variance of the first common factor was 34.3% for the FP, 24.6% for the G-8, and 19.3% for the 32-item FI; all values were below the 40% threshold. Meanwhile, their Cronbach’s α coefficients were 0.726 (FP), 0.824 (G-8), and 0.873 (32-item FI), all exceeding the acceptable threshold of 0.7. The split-half reliability coefficients were 0.614 (FP), 0.743 (G-8), and 0.763 (32-item FI), respectively.

All frailty assessments were performed by trained research staff within 3 to 5 days after hospital admission for elective surgery. Before patient recruitment, the research team formulated standardized administration protocols to guarantee uniform evaluation procedures across all participants. All patients and their caregivers received thorough explanations regarding the study objectives, clinical significance, and assessment workflow, and written informed consent was acquired from every participant prior to enrollment.

All questionnaires were completed via face-to-face interviews. Research staff read each scale item aloud, and patients provided responses with assistance from their family members where available. During sequential administration of the four frailty tools, questions addressing overlapping conceptual domains were posed only once to participants to reduce researcher burden and minimize bias related to recorded completion time. Items evaluating unintentional weight loss, physical mobility, and exhaustion overlapped across the FP, G-8, and 32-item FI. Domains covering nutritional status, cognitive function, and self-perceived health were shared between the G-8 and the 32-item FI. The CFS adopts holistic clinical assessment without separate questionnaire items, so no overlapping survey questions exist for this tool. For participants without accompanying caregivers, trained researchers guided them to recall relevant health information to complete the survey. Additional clarification was offered whenever patients failed to fully comprehend individual items. All assessment data were documented uniformly by investigators on-site. Daily supervision was performed by an independent investigator, who routinely cross-checked the completeness and accuracy of all collected data to ensure overall data validity. Periodic data auditing was conducted throughout the study period, and all incomplete or ambiguous questionnaires were centrally reviewed and standardizedly corrected to minimize potential measurement bias.

### Data collection and patient follow‑up

Data on frailty assessment scores and the time required for each tool were recorded. Additional data were collected using standardized forms, encompassing the following five categories: preoperative characteristics, surgical and pathological findings, postoperative complications, recovery status (including time to first flatus, time to liquid diet initiation, and length of postoperative hospital stay), and follow-up information. Overall postoperative complications were defined as Clavien–Dindo grade I to V, while postoperative clinically relevant complications were defined as grade ≥II (26). Postoperative telephone follow-ups were conducted every three months to assess survival through the 12-month study period. The last follow-up date in this study was December 31, 2025.

### Statistical analysis

Statistical analyses were performed using R software (version 4.4). Normally distributed data were presented as mean ± standard deviation (SD), and non-normally distributed data as median [interquartile range (IQR)]. Cohen's weighted kappa coefficient was employed to assess agreement between different frailty assessment scales. Cronbach’s alpha and split-half reliability were computed to evaluate the internal consistency of each scale, and the common latent factor method was adopted to test potential common method bias. Multivariable logistic regression and Cox regression were used to identify risk factors for clinically relevant postoperative complications and 1-year postoperative mortality, respectively. Backward stepwise selection based on the Akaike information criterion (AIC) was applied for variable selection. Candidate variables included those with a univariable P value < 0.10 and those considered clinically relevant; these variables were included in the multivariable analysis. To assess potential selection bias arising from missing frailty assessment data, we conducted a sensitivity analysis using multiple imputation. A two-sided p-value < 0.05 was considered statistically significant for all tests.

## Results

### Patient clinical characteristics

From June 2022 to December 2024, a total of 1,096 patients were included in the primary analysis. After screening and exclusions, 615 patients were finally included in the study. Baseline characteristics of patients included in the final study cohort were presented in **Table 1**. The mean patient's age was 72.6 years ± 5.5 (range, 65–91 years), with 63.9% aged 70 years or older. The cohort comprised 54.3% males and 61.8% colon cancer cases. The overall and major postoperative complication rates were 36.7% and 27.8%, respectively. And, 99 (16.1%) patients died within one year after surgery. The details of the postoperative complications and postoperative recovery of the cohort were shown in **Supplementary Table 1**. Significant differences were observed in age, BMI, pathological TNM stage, and the 1-year postoperative mortality rate between patients who completed all four frailty assessment scales and those who did not (**Supplementary Table 2**).

**Table 1:** Demographic and other characteristics of 615 older patients with colorectal cancer.

Parameter	Overall cohort (n=615)
Age, years, $\bar{x}$ ± *s*	72.6 ± 5.5
65-69	222 (36.1)
70-74	184 (29.9)
75-79	129 (21.0)
80+	80 (13.0)
Gender, n (%)	
Male	334 (54.3)
Female	281 (45.7)
BMI, kg/m^2^, $\bar{x}$ ± *s*	23.9 ± 3.7
ASA score, n (%)	
I-II	354 (57.6)
III-IV	261 (42.4)
Previous abdominal surgery, n (%)	127 (20.7)
Neoadjuvant therapy, n (%)	49 (8.0)
Tumor location	
Colon	380 (61.8)
Rectum	235 (38.2)
Surgical approach, n (%)	
Minimally invasive surgery	536 (87.2)
Open abdominal surgery	79 (12.8)
Pathological TNM stage, n (%)	
I	170 (27.6)
II	207 (33.7)
III	188 (30.6)
IV	50 (8.1)
Medical Center, n (%)	
Xi'an cohort	497 (80.8)
Yulin cohort	118 (19.2)
Postoperative overall complications, n (%)	226 (36.7)
Postoperative clinically relevant complications, n (%)	171 (27.8)
Death within one year after surgery, n (%)*	99 (16.1)

*Clavien-Dindo classification ≥ II.

### Feasibility and outcomes of the four frailty assessment tools for older patients with CRC

A total of 1,096 patients were enrolled and completed at least one frailty assessment. The respective effective response rates for the CFS, FP, G-8, and 32-item FI were 91.1%, 65.7%, 71.6%, and 58.3%. Notably, response rates for all tools exhibited a gradual decline with increasing patient age. The median completion times for the four frailty scales were 6 (ranging from 3 to 15), 10 (ranging from 6 to 22), 8 (ranging from 4 to 19), and 18 (ranging from 10 to 35) minutes, respectively (**Supplementary Table 3**).

A total of 615 patients who completed all four frailty assessments constituted the final analytic cohort. **Figure 2** displayed the assessment results across age subgroups. The prevalence of frailty was 27.0% (n=166) with CFS, 23.6% (n=145) with FP, 33.0% (n=203) with G-8, and 36.9% (n=227) with the 32-item FI. Correspondingly, the prevalence of pre-frailty was 61.8% (n=380), 55.3% (n=340), 35.6% (n=219), and 33.0% (n=203) for each tool, respectively. The scores for each age subgroup are detailed in **Supplementary Table 4**. Agreement among the scales, assessed by weighted kappa coefficients, is presented in **Table 2**. The results indicated moderate overall agreement, with the highest consistency observed between the 32-item FI and the G-8 scale (weighted kappa = 0.576).

**Figure 2:**
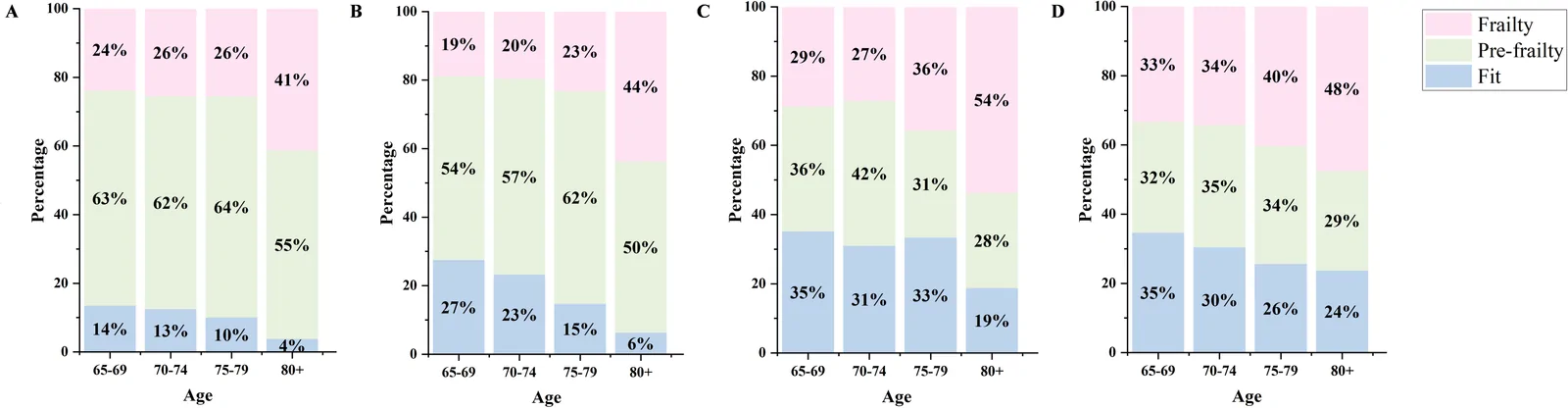
Distribution of frailty categories as assessed by the four frailty scales: **(A)** Clinical Frailty Scale; **(B)** Frailty Phenotype; **(C)** Geriatric-8; **(D)** 32-item Frailty Index.

**Table 2:** Kappa coefficient analysis among four frailty assessment tools.

Scales	G-8	FP	32-item FI	CFS
G-8	–			
FP	0.398	–		
32-item FI	0.576	0.548	–	
CFS	0.362	0.389	0.356	–

CFS, Clinical Frailty Scale; FP, frailty phenotype; G-8, Geriatric-8; FI, frailty index.

The overlap among the four frailty classifications is depicted in **Figure 3**. An agreement between instruments was identified for 83 (13.5%) participants classified as frail by all tools and 332 (53.9%) uniformly classified as non-frail. As shown in the forest plot (**Figure 4**), age, BMI, and M1 stage were risk factors for the occurrence of frailty in older CRC patients. Furthermore, preoperative frailty status, as defined by the four scales, was significantly associated with differences in postoperative complications and recovery (**Supplementary Tables 5**-**8**).

**Figure 3:**
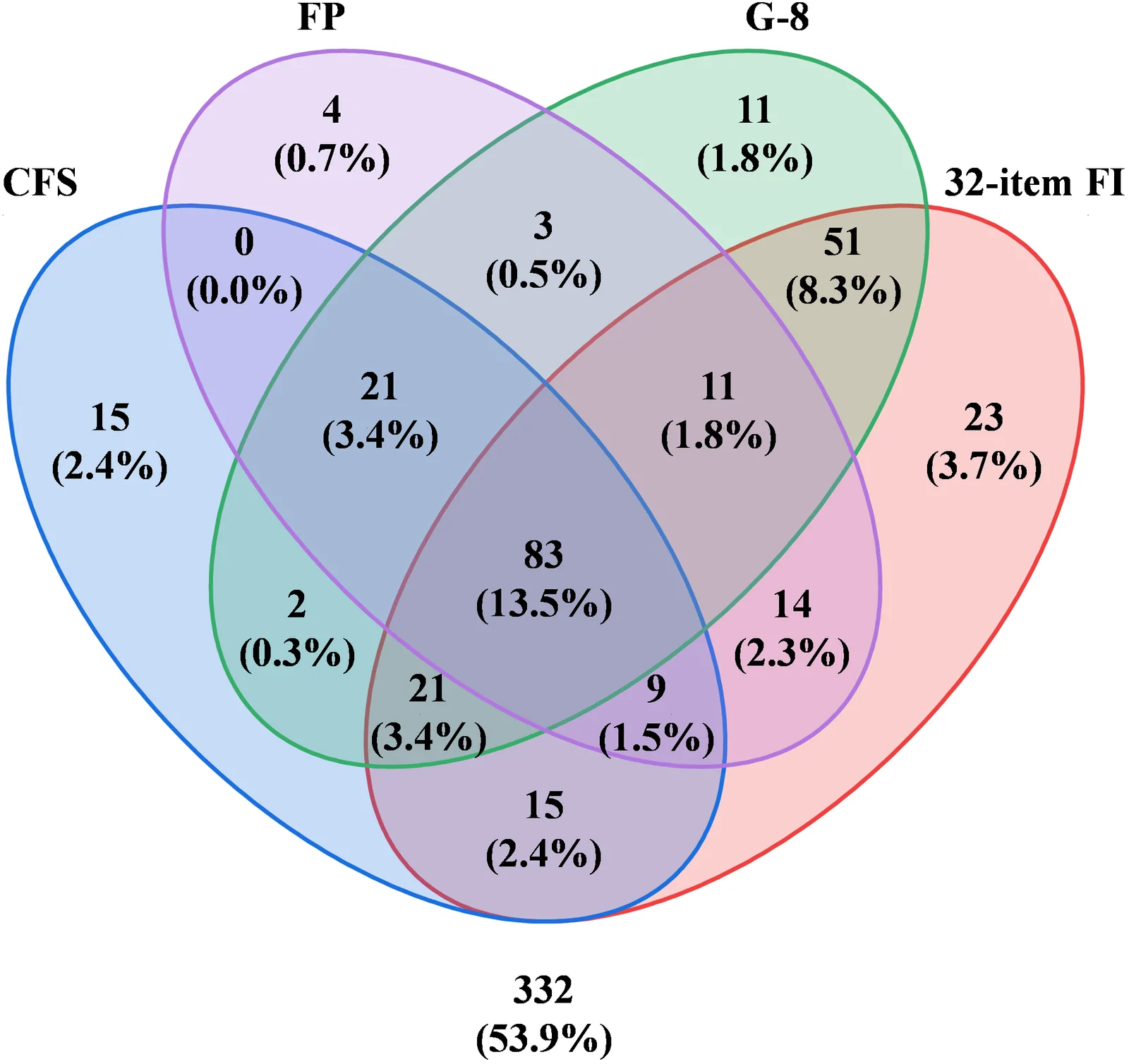
Venn diagram representing the overlap between the four frailty classifications for older patients with colorectal cancer. CFS, clinical frailty scale; FP, frailty phenotype; G-8, geriatric-8; FI, frailty index.

**Figure 4:**
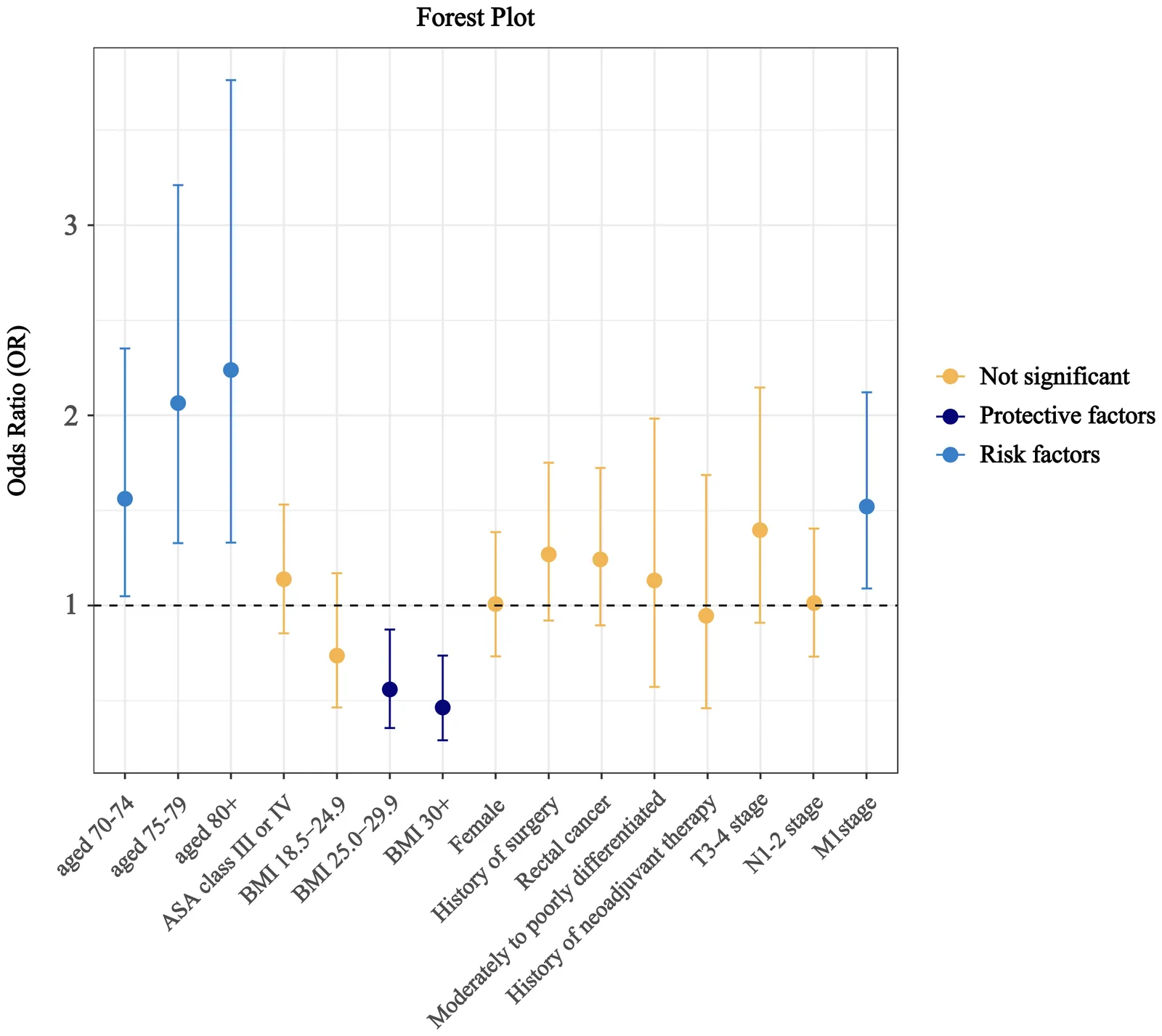
Forest plot of risk factors for frailty in older patients with colorectal cancer.

### Risk factors for postoperative clinically relevant complications in older patients with CRC

**Table 3** presented the results of univariate and multivariate logistic regression analyzes for postoperative clinically relevant complications. In univariate analysis, age, surgical approach, clinical T stage, and all four frailty scores were significantly associated with increased risk of postoperative clinically relevant complications. And, the 32-item FI yielded the highest AUC value (0.663, 95%CI: 0.624-0.700) for postoperative clinically relevant complications when compared with the other three frailty tools (**Supplementary Figure 1**). Multicollinearity was assessed prior to multivariable analysis. All four frailty scales exhibited tolerance values greater than 0.479 and variance inflation factors below 2.087, indicating that collinearity was not severe and that all variables were suitable for inclusion in the multivariable model. Subsequent multivariate analysis demonstrated that age ≥80 years (OR = 3.160, 95%CI: 1.714-5.825, *P* = 0.002), surgical approach (minimally invasive surgery, OR = 4.018, 95%CI: 2.373-6.804, p<0.001), clinical T stage (T3-4, OR = 1.718, 95%CI: 1.114-2.649, *P* = 0.014), G-8 score (frailty, OR = 2.412, 95%CI: 1.300-4.474, *P* = 0.008), and 32-item FI-defined frailty (OR = 3.035, 95%CI: 1.359-6.779, *P* = 0.017) were independent risk factors for postoperative clinically relevant complications in older patients with CRC.

**Table 3:** Univariate and multivariate analysis of risk factors for postoperative clinically relevant complications in older patients with colorectal cancer.

Variables	Univariate analysis		Multivariate analysis	
	Odds ratio (95 CI)	*p*-value	Odds ratio (95 CI)	*p*-value
Age		<0.001		0.002
65-69	Reference		Reference	
70-74	1.509 (0.950-2.398)		1.363 (0.819-2.266)	
75-79	1.818 (1.105-2.992)		1.935 (1.118-3.347)	
80+	3.481 (2.009-6.031)		3.160 (1.714-5.825)	
Sex		0.485		
male	Reference			
female	0.882 (0.619-1.255)			
BMI		0.237		
<18.5	Reference			
18.5～24.9	0.950 (0.571-1.578)			
25.0～29.9	0.789 (0.470-1.324)			
≥30	1.313 (0.801-2.151)			
ASA score		0.323		
I or II	Reference			
III or IV	1.196 (0.838-1.707)			
Tumor size, mm		0.784		
<40	Reference			
>40	1.051 (0.738-1.497)			
History of abdominal surgery		0.207		
No	Reference			
Yes	1.313 (0.860-2.003)			
History of smoking		0.806		
No	Reference			
Yes	1.052 (0.702-1.577)			
History of drinking		0.436		
No	Reference			
Yes	1.203 (0.755-1.918)			
Tumor location		0.498		
Colon	Reference			
Rectum	1.133 (0.790-1.625)			
Differentiation grade		0.369		
Well or moderate	Reference			
Poor or worse	1.227 (0.786-1.915)			
Surgical approach		<0.001		<0.001
Minimally invasive surgery*	Reference		Reference	
Open surgery	4.880 (2.984-7.982)		4.018 (2.373-6.804)	
Clinical T stage		0.048		0.014
T1-2	Reference		Reference	
T3-4	1.472 (1.004-2.160)		1.718 (1.114-2.649)	
Clinical N stage		0.974		
N0	Reference			
N1-2	1.006 (0.700-1.445)			
Neoadjuvant therapy		0.149		
No	Reference			
Yes	1.567 (0.852-2.884)			
CFS score		<0.001		0.908
Fit	Reference		Reference	
Pre-frailty	1.260 (0.658-2.412)		1.173 (0.554-2.483)	
Frailty	3.300 (1.677-6.493)		1.184 (0.518-2.706)	
FP score		<0.001		0.818
Fit	Reference		Reference	
Pre-frailty	1.703 (1.004-2.888)		0.824 (0.390-1.743)	
Frailty	4.336 (2.452-7.668)		0.957 (0.410-2.237)	
G-8 score		<0.001		0.008
Fit	Reference		Reference	
Pre-frailty	1.352 (0.816-2.240)		0.959 (0.512-1.798)	
Frailty	4.597 (2.865-7.376)		2.412 (1.300-4.474)	
32-FI score		<0.001		0.017
Fit	Reference		Reference	
Pre-frailty	2.753 (1.601-4.735)		2.978 (1.302-6.810)	
Frailty	5.237 (3.121-8.787)		3.035 (1.359-6.779)	

ASA, American Society of Anesthesiologists physical status classification; *laparoscopic surgery or robotic surgery; CFS, Clinical Frailty Scale; FP, frailty phenotype; G-8, Geriatric-8; FI, frailty index.

### Survival risk analysis within one year after surgery based on different frailty assessment tools

As illustrated in **Figure 5**, all four frailty assessment tools (CFS, FP, G-8, and 32-item FI) revealed statistically significant differences in one-year survival rates among fit, pre-frail, and frail older CRC patients. The 1-year survival rates for fit, pre-frail, and frail patients were as follows: CFS (81.2% vs. 87.9% vs. 75.9%, *P* < 0.05); FP (95.4% vs. 83.8% vs. 73.8%, *P* < 0.05); G-8 (86.5% vs. 90.0% vs. 74.9%, *P* < 0.05); and 32-item FI (93.0% vs. 88.2% vs. 72.7%, *P* < 0.05).

**Figure 5:**
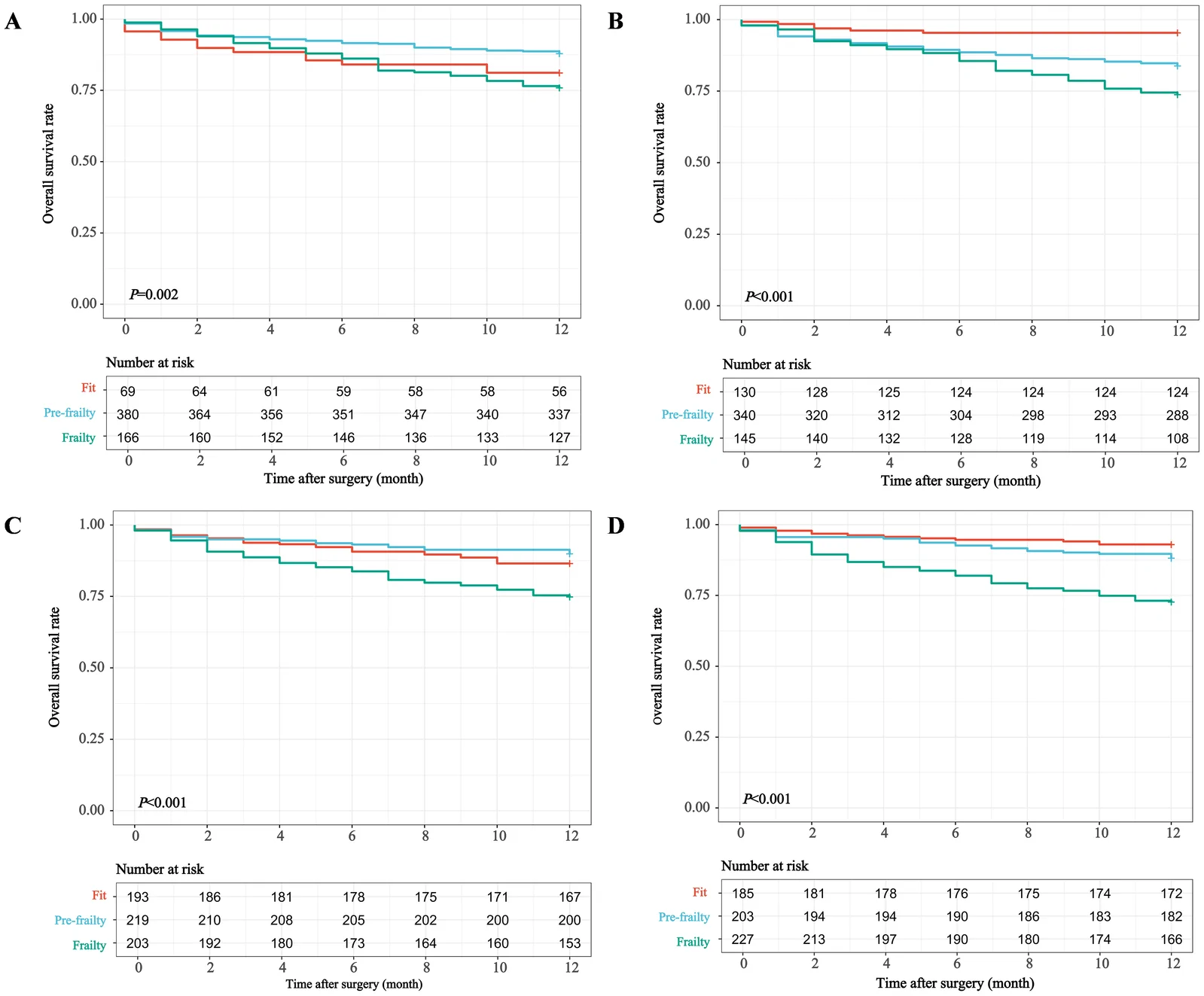
Kaplan-Meier curve of 1-year all-cause mortality of older patients with colorectal cancer according to different frailty categories: **(A)** Clinical Frailty Scale; **(B)** Frailty Phenotype; **(C)** Geriatric-8; **(D)** 32-item Frailty Index.

### Risk factors for 1-year all-cause mortality in older patients with CRC

The univariate and multivariate Cox regression analysis of risk factors for 1-year mortality was shown in **Table 4**. Univariate Cox regression results showed that age, pathological T stage, pathological N stage, pathological M stage, CFS score, FP score, G8 score, and 32-item FI score were associated with mortality within 1-year after surgery. Multivariate analysis revealed that age (≥80 years, HR = 3.427, 95%CI: 1.754-6.693, *P* = 0.003), pathological T stage (T4 stage, HR = 2.138, 95%CI: 0.910-5.023, *P* < 0.001), pathological M stage (M1 stage, HR = 3.294, 95%CI: 2.017-5.381, *P* < 0.001), and 32-item FI (frailty, HR = 2.777, 95%CI: 1.241-6.218, *P* = 0.020) were independent prognostic risk factors for 1-year all-cause mortality in older patients with CRC. Time-dependent receiver operating characteristic curves further demonstrated that the 32-item FI achieved the highest predictive performance for 1-year postoperative all-cause mortality (**Supplementary Figure 2**). However, its apparent superiority over other frailty assessment tools did not reach statistical significance in all pairwise comparisons (**Supplementary Tables 10**, **11**). Sensitivity analyses using multiple imputation for missing frailty assessment data and multivariable covariate adjustment confirmed the robustness of our findings (**Supplementary Tables 12**–**15**).

**Table 4:** Univariate and multivariate analysis of risk factors for one-year mortality in older patients with colorectal cancer.

Variables	Univariate analysis			Multivariate analysis	
	HR (95% CI)		*p*-value	HR (95% CI)	*p*-value
Age		0.006			0.003
65-69	Reference			Reference	
70-74	1.502 (0.865-2.608)			1.501 (0.845-2.668)	
75-79	2.440 (1.417-4.201)			1.870 (1.042-3.357)	
80+	2.329 (1.257-4.317)			3.427 (1.754-6.693)	
Sex		0.850			
male	Reference				
female	1.039 (0.700-1.542)				
BMI		0.319			
<18.5	Reference				
18.5∼24.9	0.614 (0.351-1.072)				
25.0∼29.9	0.676 (0.393-1.164)				
≥30	0.765 (0.450-1.301)				
ASA score		0.513			
I or II	Reference				
III or IV	1.141 (0.768-1.695)				
Tumor size, mm		0.607			
<40	Reference				
>40	0.902 (0.608-1.337)				
History of abdominal surgery		0.257			
No	Reference				
Yes	1.257 (0.847-1.864)				
History of smoking		0.984			
No	Reference				
Yes	1.005 (0.639-1.581)				
History of drinking		0.380			
No	Reference				
Yes	1.251 (0.759-2.063)				
Tumor location		0.909			
Colon	Reference				
Rectum	1.024 (0.683-1.535)				
Medical Center		0.296			
Xi'an cohort	Reference				
Yulin cohort	0.742 (0.428-1.285)				
Differentiation grade		0.444			
Well or moderate	Reference				
Poor or worse	1.207 (0.746-1.954)				
Surgical approach		0.659			
Minimally invasive surgery*	Reference				
Open surgery	1.136 (0.645-1.999)				
Pathological T stage		<0.001			0.022
T1	Reference			Reference	
T2	0.733 (0.283-1.990)			0.751 (0.285-1.983)	
T3	1.647 (0.784-3.460)			1.107 (0.510-2.403)	
T4	3.995 (1.802-8.680)			2.138 (0.910-5.023)	
Pathological N stage		<0.001			0.477
N0	Reference			Reference	
N1	1.329 (0.810-2.181)			0.965 (0.569-1.638)	
N2	2.732 (1.708-4.372)			1.338 (0.774-2.313)	
Pathological M stage		<0.001			<0.001
M0	Reference			Reference	
M1	4.691 (2.977-7.393)			3.294 (2.017-5.381)	
Neoadjuvant therapy		0.232			
Yes	Reference				
No	1.883 (0.597-5.942)				
Adjuvant therapy		0.207			
Yes	Reference				
No	1.364 (0.842-2.208)				
CFS score		0.003			0.741
Fit	Reference			Reference	
Pre-frailty	0.611 (0.330-1.130)			0.812 (0.404-1.630)	
Frailty	1.266 (0.677-2.367)			0.751 (0.360-1.565)	
FP score		<0.001			0.227
Fit	Reference			Reference	
Pre-frailty	3.702 (1.594-8.599)			2.262 (0.891-5.741)	
Frailty	6.151 (2.600-14.552)			2.250 (0.790-6.415)	
G-8 score		<0.001			0.564
Fit	Reference			Reference	
Pre-frailty	0.737 (0.417-1.300)			0.703 (0.321-1.541)	
Frailty	1.974 (1.231-3.167)			1.072 (0.575-1.996)	
FI score		<0.001			0.020
Fit	Reference			Reference	
Pre-frailty	1.715 (0.873-3.368)			1.303 (0.538-3.159)	
Frailty	4.297 (2.363-7.814)			2.777 (1.241-6.218)	

HR, hazard ratio; ASA, American Society of Anesthesiologists physical status classification; *laparoscopic surgery or robotic surgery; CFS, Clinical Frailty Scale; FP, frailty phenotype; G-8, Geriatric-8; FI, frailty index.

## Discussion

The early identification of frailty is crucial for optimizing outcomes in geriatric CRC patients. While several assessment tools have been developed and validated in this population, no single instrument has been consistently applied as a universal standard (27). To our knowledge, this is the first study to compare the four commonly used frailty scales' capacity to identify adverse clinical outcomes in older adults with CRC. The findings of our study may assist clinicians in selecting context-appropriate frailty tools based on patient characteristics and clinical constraints while being safe and effective.

The reported prevalence of frailty in CRC patients varies globally, largely due to differences in assessment methods, with estimates ranging from 12% to 76% (28, 29). In this study, the prevalence defined by four frailty scales ranged from 23.6% to 36.9%. Although the study enrolled patients aged 65 years or older, the comparatively lower prevalence observed in our cohort may be explained by the refined stratification of patients into frail, pre-frail, and fit categories. Our results showed that the 32-item FI classification can identify more frail patients than the other three methods. This finding may be attributable to the exclusion of items reflecting social and psychological deficits in the other three frailty assessment tools. In addition, this study identified advanced age, low BMI, and M1 stage as risk factors for frailty among older patients with CRC. Consistent with our findings, Zhang et al. similarly reported that advanced age, poor handgrip strength, lack of regular physical activity, and reduced nutritional scores correlated with higher frailty risk in their study population (30). Collectively, these data highlight that advanced age, impaired nutritional status, and tumor metastatic burden are universal vulnerability markers for frailty across older adults with gastrointestinal malignancies. For patients presenting with any of these risk factors upon hospital admission, clinicians should prioritize standardized preoperative frailty screening. Early recognition of frailty risk further enables personalized perioperative interventions, including nutritional support, physical prehabilitation, and targeted geriatric management, to mitigate frailty-related postoperative adverse events.

In this study, the frailty status based on four different frailty assessment tools was significantly associated with an increased incidence of postoperative complications and a prolonged recovery period in older patients with CRC. In a meta-analysis that included 14 studies, it was confirmed that the presence of frailty was significantly associated with an increased risk of postoperative complications in older patients with colon cancer (31). Our results were identical to those reported in previous studies. Frailty represents a spectrum of systemic decline and reduced homeostatic reserve associated with aging. The four frailty assessment scales in this study assessed multiple domains—including functional status, malnutrition, comorbidities, and cognitive and psychosocial factors—all of which are known to correlate with adverse perioperative outcomes in CRC patients. However, a key unresolved question is whether frailty itself carries independent prognostic value or merely reflects the integrated impact of diminished functional and nutritional reserves on surgical stress tolerance. Clarifying this distinction represents an important direction for future research.

A multicenter study of 9,397 CRC patients revealed no significant difference in cancer-specific survival between elderly and younger patients who survived beyond one year, suggesting that the observed survival disadvantage in older adults is primarily attributable to early mortality (32). This underscores the critical importance of perioperative care and first-year management. Accordingly, we conducted a follow-up study to compare the ability of different frailty assessment tools to discriminate all-cause mortality within one year after surgery. To our knowledge, this is the first study to directly compare the discriminatory efficacy of multiple frailty instruments for predicting one-year mortality in older patients following CRC surgery. Our results demonstrated that all four frailty tools served as significant predictors of one-year all-cause mortality. Notably, after adjusting for multiple confounders, only the 32-item FI remained an independent risk factor in this patient population. These findings are similar to those reported by Shang et al., who identified the American Society of Anesthesiologists grades as an independent risk factor for one-year postoperative mortality in older CRC patients, while TNM stage showed no significant association (33). The concordance between these studies underscores the clinical relevance of assessing and managing postoperative frailty in this population. Although the TNM staging system has been widely regarded as the gold standard for prognostic prediction in CRC over the past decades, its limited impact on short-term prognosis in older patients may be explained by the fact that these individuals are more likely to die from age-related chronic conditions rather than from cancer itself. Therefore, in formulating postoperative treatment plans for older CRC patients, it is essential to consider not only tumor stage but also to incorporate systematic management of frailty status.

A comprehensive frailty assessment should systematically evaluate multiple domains influencing physiologic reserve—such as comorbidities, nutritional status, and functional capacity—to identify individuals with heightened vulnerability to stressors. Furthermore, an effective frailty tool must integrate discriminative accuracy, prognostic value for clinical outcomes, and practical applicability in routine care settings. In this study, the 32-item FI demonstrated the highest predictive power for adverse outcomes in older CRC patients. However, its median completion time of 18 minutes and maximum time of 35 minutes reveal critical shortcomings that restrict routine clinical application. Its 32 multi-system deficit items bring heavy time costs to ward clinicians, require rigorous standardized training for assessors, and cannot meet the demand of rapid preoperative risk screening in busy general surgery departments, resulting in poor generalizability in daily practice. Therefore the 32-item FI is more suitable for clinical research and comprehensive individualized geriatric evaluation among high-risk patients. The FP, while widely cited, assesses frailty primarily from a physiological perspective and incorporates performance measures (e.g., grip strength, walking speed) that may be impractical or unfeasible in acutely ill or functionally impaired patients. In contrast, the CFS offers high clinical applicability due to its simplicity, though its reliance on clinician judgment may introduce subjectivity. It remains valuable when patient cooperation is limited or other tools cannot be administered. The G-8 scale components include five main domains that account for comorbidities, daily activities, health attitudes, functionality, and nutrition. In this study, the G-8 scale has high feasibility and consistency with the 32-item FI (kappa=0.576). These characteristics position the G-8 as a practical alternative when assessment time is limited or when patients cannot complete performance-based items. Frailty evaluation constitutes merely one component of comprehensive GA, and the multi-domain frailty tools adopted in the present study may still possess certain deficiencies. Whether combining multiple complementary single-dimensional screening items can accomplish complete GA more efficiently, cut down assessment duration, and better adapt to routine preoperative clinical implementation remains a critical question worthy of further exploration, representing a key direction for subsequent research. Additionally, a mature, standardized full GA evaluation system requires external validation across diverse patient cohorts, which is another promising avenue for future investigations.

Frailty assessment embedded within comprehensive GA enables accurate risk stratification, which further facilitates the formulation of personalized frailty management strategies for vulnerable patients. Prehabilitation for oncological populations generally adopts a multimodal, multidisciplinary model that integrates physical exercise training, nutritional optimization, psychological support, and smoking and alcohol cessation counselling. Existing observational and prospective cohort studies have consistently demonstrated that prehabilitation programs effectively lower the incidence of postoperative complications and accelerate functional recovery relative to standard routine care (34, 35). In our cohort, impaired mobility and malnutrition were prevalent among most frail patients with CRC. Optimizing physical function enables early postoperative mobilization, which accelerates the recovery of gastrointestinal motility and overall rehabilitation. Likewise, preoperative nutritional optimization strengthens patients’ physiological reserve, alleviates surgical metabolic stress, and lowers the risk of perioperative complications (36). Therefore, physical and nutritional interventions ought to serve as core components when designing prehabilitation protocols for older CRC patients. Intraoperative refined strategies—including goal-directed fluid resuscitation, individualized anesthetic regimens, and meticulous minimally invasive surgical techniques—can also further decrease the occurrence of adverse perioperative events. In addition, one prospective cohort study demonstrated that sustained aerobic exercise yields the most prominent improvements in disease-free survival among patients with advanced colon cancer (37). Taken together, personalized full-cycle frailty management stratified by baseline frailty status, encompassing preoperative prehabilitation, intraoperative precision care, and consistent postoperative rehabilitation, represents a promising research avenue for improving long-term clinical outcomes in older CRC populations.

This multicenter prospective cohort study has several noteworthy limitations. First, as an observational design, our analysis only identifies correlational relationships rather than causal effects; accordingly, causal inferences drawn from these results should be interpreted with great caution. Second, our final analytical sample was restricted to participants completing all four frailty assessments. This inclusion rule excluded critically ill, cognitively impaired, physically debilitated, or poorly cooperative patients who could not undergo the lengthy multi-tool evaluation; such patients may have carried a higher frailty burden, which could potentially attenuate or inflate the observed associations. While our sensitivity analyses support the robustness of results, residual selection bias cannot be completely excluded. This limits external validity, and our findings may not apply to all older patients with CRC. Developing tailored frailty assessment strategies for these vulnerable, hard-to-assess subgroups warrants further study. Third, our follow-up duration was limited to one year, which prevents us from evaluating long-term oncologic outcomes. Extended longitudinal monitoring is required to further clarify the long-term prognostic predictive value of the four frailty tools among older CRC patients. Finally, we stratified participants into fit, pre-frail and frail groups to clearly describe frailty distribution. However, the diagnostic standards for pre-frailty have not been sufficiently validated by consistent evidence. Thus, we highlight that further research is required to optimize pre-frail cutoff values and quantify the predictive effect of pre-frailty on postoperative adverse outcomes.

## Conclusion

In conclusion, all four frailty assessment methods demonstrated significant associations with adverse outcomes in geriatric patients with CRC. Regardless of the tool used, patients identified as frail experienced a substantially higher incidence of postoperative complications, prolonged recovery, and increased one-year all-cause mortality. In our specific cohort, the 32-item FI demonstrated relatively higher discriminative ability for predicting postoperative clinically relevant complications and one-year mortality; however, its clinical feasibility was limited due to a longer administration time. These findings underscore the importance of selecting an appropriate frailty assessment strategy based on specific clinical contexts and individual patient characteristics.

## Data Availability

The raw data supporting the conclusions of this article will be made available by the authors, without undue reservation.
