# Supplementary material for: Comparison of feasibility, inter-instrument agreement, and prognostic value of four frailty assessment instruments in geriatric colorectal cancer patients: a 3-year multicenter prospective cohort study
Source: Front Oncol. 2026 Oct 9;16:1878424. doi: 10.3389/fonc.2026.1878424 (PMC13690656; doi:10.3389/fonc.2026.1878424)
Supplement: Supplementary file 1 [file SupplementaryFile1.docx]

**Comparison of feasibility, inter-instrument agreement, and prognostic value of four frailty assessment instruments in geriatric colorectal cancer patients: a 3-year multicenter prospective cohort study**

Ma et al.

(Supplementary materials)

**Supplementary Table 1.** Postoperative recovery profiles and postoperative complications of 615 older patients with colorectal cancer

| Parameter | (n=615) |
| --- | --- |
| Postoperative overall complications, n (%) | 226 (36.7) |
| Postoperative major complications, n (%)^*^ | 171 (27.8) |
| Wound infection | 51 (8.3) |
| Intestinal obstruction | 32 (5.2) |
| Anastomotic leakage | 21 (3.4) |
| Anastomotic bleeding | 27 (4.4) |
| Intra-abdominal abscess | 14 (2.3) |
| Pulmonary infection | 43 (7.0) |
| Cardiovascular and cerebrovascular complications | 4 (0.7) |
| Urinary tract infection | 12 (2.0) |
| Urinary retention | 19 (3.1) |
| Stoma-related complications | 9 (1.5) |
| Intestinal fistula | 7 (1.1) |
| Days to first flatus (days), median (IQR) | 2 (1-3) |
| Days to soft diet (days), median (IQR) | 3 (2-5) |
| Postoperative hospital stay (days), median (IQR) | 12 (9-14) |
| 30-day readmission, n (%) | 91 (14.8) |
| ^*^, Clavien-Dindo classification ≥ II; IQR, interquartile range. | |

**Supplementary Table 2. Comparison of clinicopathological characteristics between patients who completed all frailty assessment scales and those who did not**

| Parameter | Study cohort  (n=615) | Excluded cohort  (n=452) | *P*-value |
| --- | --- | --- | --- |
| Age, years, $\bar{x}$±*s* | 72.6±5.5 | 74.7±6.7 | <0.001 |
| Gender, n (%) |  |  | 0.183 |
| Male | 334 (54.3) | 264 (58.4) |  |
| Female | 281 (45.7) | 188 (41.6) |  |
| BMI, kg/m^2^, $\bar{x}$±*s* | 23.9±3.7 | 22.4±4.1 | <0.001 |
| ASA score, n (%) |  |  | <0.001 |
| I-II | 354 (57.6) | 212 (46.9) |  |
| III-IV | 261 (42.4) | 240 (51.4) |  |
| Previous abdominal surgery, n (%) | 127 (20.7) | 101 (22.3) | 0.505 |
| Neoadjuvant therapy, n (%) | 49 (8.0) | 46 (10.2) | 0.210 |
| Tumor location |  |  | 0.160 |
| Colon | 380 (61.8) | 260 (57.5) |  |
| Rectum | 235 (38.2) | 192 (42.5) |  |
| Surgical approach, n (%) |  |  | 0.303 |
| Minimally invasive surgery | 536 (87.2) | 384 (85.0) |  |
| Open abdominal surgery | 79 (12.8) | 68 (15.0) |  |
| Pathological TNM stage, n (%) |  |  | 0.001 |
| I | 170 (27.6) | 81 (17.9) |  |
| II | 207 (33.7) | 154 (34.1) |  |
| III | 188 (30.6) | 166 (36.7) |  |
| IV | 50 (8.1) | 51 (11.3) |  |
| Postoperative overall complications, n (%) | 226 (36.7) | 191 (42.3) | 0.068 |
| Postoperative clinically relevant complications, n (%)^*^ | 171 (27.8) | 148 (32.7) | 0.082 |
| Death within one year after surgery, n (%) | 99 (16.1) | 104 (23.0) | 0.004 |
| ^*^, Clavien-Dindo classification ≥ II | |  |  |

**Supplementary Table 3.** Feasibility of four frailty assessment tools in older colorectal cancer patients according to different ages

| Scale | 65-69 group  (n=330) | |  | 70-74 group  (n=296) | |  | 75-79 group  (n=273) | |  | 80+ group  (n=197) | |
| --- | --- | --- | --- | --- | --- | --- | --- | --- | --- | --- | --- |
|  | Feasibility, % | Time* |  | Feasibility, % | Time* |  | Feasibility, % | Time* |  | Feasibility, % | Time* |
| FP | 248 (75.2) | 9 (7-12) |  | 213 (72.0) | 10 (8-12) |  | 164 (60.1) | 11 (9-14) |  | 95 (48.2) | 13 (10-15) |
| 32-FI | 228 (69.1) | 15 (12-19) |  | 194 (65.5) | 16 (12-20) |  | 133 (48.7) | 18 (13-21) |  | 84 (42.6) | 20 (16-24) |
| G-8 | 270 (81.8) | 8 (6-10) |  | 235 (79.3) | 8 (6-11) |  | 177 (64.8) | 9 (7-11) |  | 103 (52.3) | 10 (8-12) |
| CFS | 313 (94.8) | 6 (4-8) |  | 272 (91.9) | 6 (5-8) |  | 245 (89.7) | 7 (5-9) |  | 169 (85.8) | 7 (5-10) |
| ^*^, min, median (interquartile range); CFS, clinical frailty scale; FP, frailty phenotype; G-8, Geriatric-8; FI, frailty index. | | | | | | | | | | | |

**Supplementary Table 4.** Distribution of scores from four frailty assessment tools by age group in older patients with colorectal cancer

| Scale | Overall score | 65-69 group  (n=222) | 70-74 group  (n=184) | 75-79 group  (n=129) | 80+ group  (n=80) |
| --- | --- | --- | --- | --- | --- |
| G-8, $\bar{x}$±*s* | 11.40±3.96 | 11.45±3.99 | 11.95±3.59 | 11.32±4.30 | 10.15±3.85 |
| FP, $\bar{x}$±*s* | 1.75±1.46 | 1.57±1.43 | 1.65±1.37 | 1.81±1.46 | 2.43±1.52 |
| 32-FI, $\bar{x}$±*s* | 0.24±0.16 | 0.23±0.16 | 0.22±0.14 | 0.26±0.17 | 0.27±0.16 |
| CFS, $\bar{x}$±*s* | 3.93±1.45 | 3.81±1.44 | 3.81±1.45 | 3.95±1.41 | 4.48±1.48 |
| CFS, clinical frailty scale; FP, frailty phenotype; G-8, Geriatric-8; FI, frailty index. | | | | | |

**Supplementary Table 5.** Perioperative outcome of older colorectal cancer patients stratified by the Clinical Frailty Scale

| Parameter | Fit  (n=69) | Pre-frailty  (n=380) | Frailty  (n=166) | *P*-value |
| --- | --- | --- | --- | --- |
| Postoperative overall complications, n (%) | 18 (26.1) | 109 (28.7) | 99 (59.6) | <0.001 |
| Postoperative clinically relevant complications, n (%)* | 13 (18.8) | 86 (22.6) | 72 (42.4) | <0.001 |
| Days to first flatus (days), median (IQR) | 2 (1-3) | 2 (1-3) | 3 (1-3) | 0.242 |
| Days to soft diet (days), median (IQR) | 2 (2-5) | 3 (2-5) | 4 (2-6) | 0.031 |
| Postoperative hospital stay (days), median (IQR) | 10 (7-13) | 12 (8-14) | 13 (9-15) | <0.001 |
| 30-day readmission, n (%) | 8 (11.6) | 53 (13.9) | 30 (18.1) | 0.334 |
| *, Clavien-Dindo classification ≥ II; IQR, interquartile range. | | | | |

**Supplementary Table 6.** Perioperative outcome of older colorectal cancer patients stratified by the Frailty Phenotype

| Parameter | Fit  (n=130) | Pre-frailty  (n=340) | Frailty  (n=145) | *P*-value |
| --- | --- | --- | --- | --- |
| Postoperative overall complications, n (%) | 29 (22.3) | 105 (30.0) | 92 (63.4) | <0.001 |
| Postoperative clinically relevant complications, n (%)* | 21 (16.2) | 84 (24.7) | 66 (45.5) | <0.001 |
| Days to first flatus (days), median (IQR) | 2 (1-3) | 2 (1-3) | 3 (1-3) | 0.124 |
| Days to soft diet (days), median (IQR) | 2 (1-3) | 3 (2-5) | 4 (2-5) | 0.007 |
| Postoperative hospital stay (days), median (IQR) | 9 (7-11) | 11 (8-14) | 13 (9-15) | <0.001 |
| 30-day readmission, n (%) | 12 (9.2) | 52 (15.3) | 27 (20.0) | 0.049 |
| *, Clavien-Dindo classification ≥ II; IQR, interquartile range. | | | | |

**Supplementary Table 7.** Perioperative outcome of older colorectal cancer patients stratified by the Geriatric-8 scale

| Parameter | Fit  (n=193) | Pre-frailty  (n=219) | Frailty  (n=203) | *P*-value |
| --- | --- | --- | --- | --- |
| Postoperative overall complications, n (%) | 39 (20.2) | 65 (29.7) | 122 (60.1) | <0.001 |
| Postoperative clinically relevant complications, n (%)* | 31 (16.1) | 45 (20.5) | 95 (46.8) | <0.001 |
| Days to first flatus (days), median (IQR) | 2 (1-3) | 2 (1-3) | 3 (1-3) | 0.079 |
| Days to soft diet (days), median (IQR) | 2 (2-5) | 3 (2-5) | 4 (2-6) | <0.001 |
| Postoperative hospital stay (days), median (IQR) | 11 (8-13) | 12 (8-14) | 13 (9-15) | <0.001 |
| 30-day readmission, n (%) | 19 (9.8) | 26 (11.9) | 46 (22.7) | <0.001 |
| *, Clavien-Dindo classification ≥ II; IQR, interquartile range. | | | | |

**Supplementary Table 8.** Perioperative outcome of older colorectal cancer patients stratified by the 32-item Frailty Index

| Parameter | Fit  (n=185) | Pre-frailty  (n=203) | Frailty  (n=227) | *P*-value |
| --- | --- | --- | --- | --- |
| Postoperative overall complications, n (%) | 26 (14.1) | 69 (34.0) | 131 (57.7) | <0.001 |
| Postoperative clinically relevant complications, n (%)* | 22 (11.9) | 55 (27.1) | 94 (41.4) | <0.001 |
| Days to first flatus (days), median (IQR) | 1 (1-3) | 2 (1-3) | 3 (1-3) | 0.014 |
| Days to soft diet (days), median (IQR) | 2 (1-3) | 3 (2-5) | 4 (2-6) | <0.001 |
| Postoperative hospital stay (days), median (IQR) | 9 (7-11) | 11 (8-14) | 13 (9-15) | <0.001 |
| 30-day readmission, n (%) | 9 (4.9) | 23 (11.3) | 59 (26.0) | <0.001 |
| *, Clavien-Dindo classification ≥ II; IQR, interquartile range. | | | | |

**Supplementary Table 9.** Separate multivariable-adjusted analyses of four frailty tools for postoperative clinically relevant complications and 1-year all-cause mortality

| Scale | | **Multivariate analysis^*^** | |  | **Multivariate analysis^&^** | |
| --- | --- | --- | --- | --- | --- | --- |
|  |  | **Odds ratio (95% CI)** | ***p*-value** |  | **Hazard ratio (95% CI)** | ***p*-value** |
| G-8 | |  | ＜0.001 |  |  | ＜0.001 |
|  | Fit | Reference |  |  | Reference |  |
|  | Pre-frailty | 1.317 (0.777-2.233) |  |  | 0.782 (0.429-1.425) |  |
|  | Frailty | 4.257 (2.585-7.008) |  |  | 2.085 (1.279-3.398) |  |
| FP | |  | ＜0.001 |  |  | ＜0.001 |
|  | Fit | Reference |  |  | Reference |  |
|  | Pre-frailty | 1.767 (1.102-3.087) |  |  | 2.919 (1.237-6.890) |  |
|  | Frailty | 4.102 (2.235-7.530) |  |  | 4.797 (2.011-11.443) |  |
| 32-FI | |  | ＜0.001 |  |  | ＜0.001 |
|  | Fit | Reference |  |  | Reference |  |
|  | Pre-frailty | 2.877 (1.624-5.097) |  |  | 1.415 (0.711-2.814) |  |
|  | Frailty | 5.364 (3.086-9.324) |  |  | 3.858 (2.090-7.121) |  |
| CFS | |  | ＜0.001 |  |  | 0.009 |
|  | Fit | Reference |  |  | Reference |  |
|  | Pre-frailty | 1.214 (0.613-2.406) |  |  | 0.583 (0.304-1.117) |  |
|  | Frailty | 2.993 (1.466-6.110) |  |  | 1.160 (0.609-2.207) |  |
| CFS, clinical frailty scale; FP, frailty phenotype; G-8, Geriatric-8; FI, frailty index.  ^*^ adjusted by age, surgical approach, and clinical T stage.  ^&^ adjusted by age, pathological T stage, pathological N stage, and pathological M stage. | | | | | | |

**Supplementary Figure 1**. AUC curves for the four frailty assessment tools predicting clinically relevant postoperative complications in elderly patients with colorectal cancer. CFS, clinical frailty scale; FP, frailty phenotype; G-8, geriatric-8; FI, frailty index.

**Supplementary Table 10**. Pairwise comparisons of AUCs of four frailty assessment tools in predicting clinically relevant postoperative complications among older patients with colorectal cancer

| Comparisons | AUC 1 | AUC 2 | *Z*-value | *P*-value |
| --- | --- | --- | --- | --- |
| 32-item FI vs. G-8 | 0.663 | 0.659 | 0.213 | 0.8314 |
| 32-item FI vs. FP | 0.663 | 0.629 | 1.341 | 0.1799 |
| 32-item FI vs. CFS | 0.663 | 0.611 | 1.961 | 0.0499 |
| G-8 vs. FP | 0.659 | 0.629 | 1.518 | 0.1291 |
| G-8 vs. CFS | 0.659 | 0.611 | 1.934 | 0.0531 |
| CFS vs. FP | 0.611 | 0.629 | 0.753 | 0.4512 |
| CFS, Clinical Frailty Scale; FP, frailty phenotype; G-8, Geriatric-8; FI, frailty index. | | | | |

**Supplementary Figure 2**. Time-dependent ROC curves of the four frailty assessment tools for predicting 1-year postoperative all-cause mortality in older patients with colorectal cancer. CFS, clinical frailty scale; FP, frailty phenotype; G-8, geriatric-8; FI, frailty index.

**Supplementary Table 11**. Pairwise comparisons of AUCs for four frailty assessment tools in predicting postoperative all-cause mortality at different time points in older patients with colorectal cancer

| Months | Comparisons | AUC 1 | AUC 2 | *Z*-value | *P*-value |
| --- | --- | --- | --- | --- | --- |
| 1 | CFS vs. FP | 0.45 | 0.534 | -1.369 | 0.171 |
| 1 | CFS vs. G-8 | 0.45 | 0.547 | -1.52 | 0.129 |
| 1 | CFS vs. 32-item FI | 0.45 | 0.605 | -2.596 | 0.009 |
| 1 | FP vs. G-8 | 0.534 | 0.547 | -0.16 | 0.873 |
| 1 | FP vs. 32-item FI | 0.534 | 0.605 | -1.454 | 0.146 |
| 1 | G-8 vs. 32-item FI | 0.547 | 0.605 | -0.875 | 0.382 |
| 2 | CFS vs. FP | 0.467 | 0.564 | -2.039 | 0.041 |
| 2 | CFS vs. G-8 | 0.467 | 0.587 | -2.116 | 0.034 |
| 2 | CFS vs. 32-item FI | 0.467 | 0.641 | -3.53 | ＜0.001 |
| 2 | FP vs. G-8 | 0.564 | 0.587 | -0.347 | 0.729 |
| 2 | FP vs. 32-item FI | 0.564 | 0.641 | -2.047 | 0.041 |
| 2 | G-8 vs. 32-item FI | 0.587 | 0.641 | -0.958 | 0.338 |
| 3 | CFS vs. FP | 0.492 | 0.562 | -1.578 | 0.115 |
| 3 | CFS vs. G-8 | 0.492 | 0.582 | -1.621 | 0.105 |
| 3 | CFS vs. 32-item FI | 0.492 | 0.658 | -3.779 | ＜0.001 |
| 3 | FP vs. G-8 | 0.562 | 0.582 | -0.312 | 0.755 |
| 3 | FP vs. 32-item FI | 0.562 | 0.658 | -2.509 | 0.012 |
| 3 | G-8 vs. 32-item FI | 0.582 | 0.658 | -1.455 | 0.146 |
| 4 | CFS vs. FP | 0.511 | 0.571 | -1.473 | 0.141 |
| 4 | CFS vs. G-8 | 0.511 | 0.594 | -1.652 | 0.098 |
| 4 | CFS vs. 32-item FI | 0.511 | 0.659 | -3.641 | ＜0.001 |
| 4 | FP vs. G-8 | 0.571 | 0.594 | -0.408 | 0.683 |
| 4 | FP vs. 32-item FI | 0.571 | 0.659 | -2.474 | 0.013 |
| 4 | G-8 vs. 32-item FI | 0.594 | 0.659 | -1.408 | 0.159 |
| 5 | CFS vs. FP | 0.511 | 0.569 | -1.559 | 0.119 |
| 5 | CFS vs. G-8 | 0.511 | 0.59 | -1.636 | 0.102 |
| 5 | CFS vs. 32-item FI | 0.511 | 0.652 | -3.59 | ＜0.001 |
| 5 | FP vs. G-8 | 0.569 | 0.59 | -0.388 | 0.698 |
| 5 | FP vs. 32-item FI | 0.569 | 0.652 | -2.535 | 0.011 |
| 5 | G-8 vs. 32-item FI | 0.59 | 0.652 | -1.388 | 0.165 |
| 6 | CFS vs. FP | 0.517 | 0.588 | -2.041 | 0.041 |
| 6 | CFS vs. G-8 | 0.517 | 0.581 | -1.399 | 0.162 |
| 6 | CFS vs. 32-item FI | 0.517 | 0.651 | -3.589 | ＜0.001 |
| 6 | FP vs. G-8 | 0.588 | 0.581 | 0.146 | 0.884 |
| 6 | FP vs. 32-item FI | 0.588 | 0.651 | -2.028 | 0.043 |
| 6 | G-8 vs. 32-item FI | 0.581 | 0.651 | -1.66 | 0.097 |
| 7 | CFS vs. FP | 0.553 | 0.608 | -1.693 | 0.091 |
| 7 | CFS vs. G-8 | 0.553 | 0.604 | -1.195 | 0.232 |
| 7 | CFS vs. 32-item FI | 0.553 | 0.665 | -3.129 | 0.002 |
| 7 | FP vs. G-8 | 0.608 | 0.604 | 0.106 | 0.916 |
| 7 | FP vs. 32-item FI | 0.608 | 0.665 | -1.917 | 0.055 |
| 7 | G-8 vs. 32-item FI | 0.604 | 0.665 | -1.578 | 0.115 |
| 8 | CFS vs. FP | 0.549 | 0.612 | -1.965 | 0.049 |
| 8 | CFS vs. G-8 | 0.549 | 0.597 | -1.164 | 0.245 |
| 8 | CFS vs. 32-item FI | 0.549 | 0.672 | -3.527 | ＜0.001 |
| 8 | FP vs. G-8 | 0.612 | 0.597 | 0.345 | 0.730 |
| 8 | FP vs. 32-item FI | 0.612 | 0.672 | -2.129 | 0.033 |
| 8 | G-8 vs. 32-item FI | 0.597 | 0.672 | -1.986 | 0.047 |
| 9 | CFS vs. FP | 0.555 | 0.623 | -2.198 | 0.028 |
| 9 | CFS vs. G-8 | 0.555 | 0.592 | -0.949 | 0.343 |
| 9 | CFS vs. 32-item FI | 0.555 | 0.668 | -3.356 | 0.001 |
| 9 | FP vs. G-8 | 0.623 | 0.592 | 0.708 | 0.479 |
| 9 | FP vs. 32-item FI | 0.623 | 0.668 | -1.62 | 0.105 |
| 9 | G-8 vs. 32-item FI | 0.592 | 0.668 | -2.082 | 0.037 |
| 10 | CFS vs. FP | 0.552 | 0.634 | -2.708 | 0.007 |
| 10 | CFS vs. G-8 | 0.552 | 0.582 | -0.798 | 0.425 |
| 10 | CFS vs. 32-item FI | 0.552 | 0.664 | -3.281 | 0.001 |
| 10 | FP vs. G-8 | 0.634 | 0.582 | 1.276 | 0.202 |
| 10 | FP vs. 32-item FI | 0.634 | 0.664 | -1.091 | 0.275 |
| 10 | G-8 vs. 32-item FI | 0.582 | 0.664 | -2.325 | 0.020 |
| 11 | CFS vs. FP | 0.563 | 0.639 | -2.561 | 0.010 |
| 11 | CFS vs. G-8 | 0.563 | 0.596 | -0.908 | 0.364 |
| 11 | CFS vs. 32-item FI | 0.563 | 0.674 | -3.356 | 0.001 |
| 11 | FP vs. G-8 | 0.639 | 0.596 | 1.081 | 0.280 |
| 11 | FP vs. 32-item FI | 0.639 | 0.674 | -1.319 | 0.187 |
| 11 | G-8 vs. 32-item FI | 0.596 | 0.674 | -2.3 | 0.021 |
| 12 | CFS vs. FP | 0.563 | 0.638 | -2.655 | 0.008 |
| 12 | CFS vs. G-8 | 0.563 | 0.596 | -0.975 | 0.330 |
| 12 | CFS vs. 32-item FI | 0.563 | 0.671 | -3.392 | 0.001 |
| 12 | FP vs. G-8 | 0.638 | 0.596 | 1.097 | 0.272 |
| 12 | FP vs. 32-item FI | 0.638 | 0.671 | -1.258 | 0.208 |
| 12 | G-8 vs. 32-item FI | 0.596 | 0.671 | -2.272 | 0.023 |
| CFS, Clinical Frailty Scale; FP, frailty phenotype; G-8, Geriatric-8; FI, frailty index. | | | | | |

**Supplementary Table 12.** Univariate and multivariate analysis of risk factors for postoperative clinically relevant complications in 1,096 older patients with colorectal cancer after multiple imputation.

| **Variables** | | **Univariate analysis** | |  | **Multivariate analysis** | |
| --- | --- | --- | --- | --- | --- | --- |
|  |  | **Odds ratio (95 %CI)** | ***p*-value** |  | **Odds ratio (95% CI)** | ***p*-value** |
| Age | |  | ＜0.001 |  |  | ＜0.001 |
|  | 65-69 | Reference |  |  | Reference |  |
|  | 70-74 | 1.530 (1.080-2.167) |  |  | 1.439 (0.973-2.127) |  |
|  | 75-79 | 1.898 (1.304-2.762) |  |  | 1.948 (1.118-3.033) |  |
|  | 80+ | 3.505 (2.323-5.290) |  |  | 3.475 (1.714-5.576) |  |
| Sex | |  | 0.518 |  |  |  |
|  | male | Reference |  |  |  |  |
|  | female | 1.092 (0.837-1.423) |  |  |  |  |
| BMI | |  | 0.025 |  |  | 0.005 |
|  | ＜18.5 | Reference |  |  | Reference |  |
|  | 18.5～24.9 | 0.950 (0.646-1.395) |  |  | 1.097 (0.699-1.720) |  |
|  | 25.0～29.9 | 0.843 (0.573-1.241) |  |  | 0.799 (0.509-1.255) |  |
|  | ≥30 | 1.441 (0.995-2.088) |  |  | 1.687 (1.086-2.618) |  |
| ASA score | |  | 0.344 |  |  |  |
|  | I or II | Reference |  |  |  |  |
|  | III or IV | 1.285 (0.765-2.158) |  |  |  |  |
| Tumor size, mm | |  | 0.490 |  |  |  |
|  | ＜40 | Reference |  |  |  |  |
|  | ＞40 | 1.098 (0.842-1.432) |  |  |  |  |
| History of abdominal surgery | |  | 0.056 |  |  | 0.023 |
|  | No | Reference |  |  | Reference |  |
|  | Yes | 1.297 (0.993-1.693) |  |  | 1426 (1.049-1.937) |  |
| History of smoking | |  | 0.582 |  |  |  |
|  | No | Reference |  |  |  |  |
|  | Yes | 1.089 (0.804-1.475) |  |  |  |  |
| History of drinking | |  | 0.396 |  |  |  |
|  | No | Reference |  |  |  |  |
|  | Yes | 1.163 (0.821-1.648) |  |  |  |  |
| Tumor location | |  | 0.459 |  |  |  |
|  | Colon | Reference |  |  |  |  |
|  | Rectum | 1.108 (0.845-1.452) |  |  |  |  |
| Medical Center | |  | 0.194 |  |  |  |
|  | Xi'an cohort | Reference |  |  |  |  |
|  | Yulin cohort | 0.794 (0.561-1.124) |  |  |  |  |
| Differentiation grade | |  | 0812 |  |  |  |
|  | Well or moderate | Reference |  |  |  |  |
|  | Poor or worse | 0.957 (0.662-1.381) |  |  |  |  |
| Surgical approach | |  | ＜0.001 |  |  | ＜0.001 |
|  | Minimally invasive surgery* | Reference |  |  | Reference |  |
|  | Open surgery | 5.048 (3.486-7.310) |  |  | 4.593 (3.040-6.940) |  |
| Clinical T stage | |  | 0.031 |  |  | 0.003 |
|  | T1-2 | Reference |  |  | Reference |  |
|  | T3-4 | 1.366 (1.028-1.815) |  |  | 1.639 (1.180-2.276) |  |
| Clinical N stage | |  | 0.944 |  |  |  |
|  | N0 | Reference |  |  |  |  |
|  | N1-2 | 0.990 (0.755-1.299) |  |  |  |  |
| Neoadjuvant therapy | |  | 0.007 |  |  | 0.021 |
|  | No | Reference |  |  | Reference |  |
|  | Yes | 1.576 (1.132-2.195) |  |  | 1571 (1.070-2.306) |  |
| CFS score | |  | ＜0.001 |  |  | 0.795 |
|  | Fit | Reference |  |  | Reference |  |
|  | Pre-frailty | 1.060 (0.658-1.708) |  |  | 1.070 (0.601-1.907) |  |
|  | Frailty | 2.911 (1.773-4.780) |  |  | 1.214 (0.643-2.293) |  |
| FP score | |  | ＜0.001 |  |  | 0.106 |
|  | Fit | Reference |  |  | Reference |  |
|  | Pre-frailty | 1.569 (1.069-2.304) |  |  | 0.550 (0.303-1.090) |  |
|  | Frailty | 3.778 (2.489-5.734) |  |  | 0.508 (0.260-1.050) |  |
| G-8 score | |  | ＜0.001 |  |  | ＜0.001 |
|  | Fit | Reference |  |  | Reference |  |
|  | Pre-frailty | 1.260 (0.864-1.837) |  |  | 0.910 (0.563-1.471) |  |
|  | Frailty | 4.5467 (3.197-6.465) |  |  | 2.181 (1.369-3.475) |  |
| FI score | |  | ＜0.001 |  |  | ＜0.001 |
|  | Fit | Reference |  |  | Reference |  |
|  | Pre-frailty | 3.195 (2.091-4.882) |  |  | 4.797 (2.463-9.342) |  |
|  | Frailty | 6.663 (4.439-10.004) |  |  | 5.517 (2.892-10.526) |  |
| ASA, American Society of Anesthesiologists physical status classification; *, laparoscopic surgery or robotic surgery; CFS, Clinical Frailty Scale; FP, frailty phenotype; G-8, Geriatric-8; FI, frailty index. | | | | | | |

**Supplementary Table 13.** Univariate and multivariate analysis of risk factors for one-year mortality in 1,096 older patients with colorectal cancer after multiple imputation.

| **Variables** | | | **Univariate analysis** | | |  | **Multivariate analysis** | |
| --- | --- | --- | --- | --- | --- | --- | --- | --- |
|  |  |  | **HR (95% CI)** | | ***p*-value** |  | **HR (95% CI)** | ***p*-value** |
| Age | | |  | 0.092 | |  |  | 0.042 |
|  | 65-69 | | Reference |  | |  | Reference |  |
|  | 70-74 | | 0.717 (0.489-1.053) |  | |  | 0.632 (0.425-0.940) |  |
|  | 75-79 | | 0.752 (0.490-1.154) |  | |  | 0.594 (0.378-0.933) |  |
|  | 80+ | | 1.204 (0.785-1.849) |  | |  | 0.935 (0.598-1.462) |  |
| Sex | | |  | 0.690 | |  |  |  |
|  | male | | Reference |  | |  |  |  |
|  | female | | 1.063 (0.787-1.435) |  | |  |  |  |
| BMI | | |  | 0.097 | |  |  | 0.199 |
|  | ＜18.5 | | Reference |  | |  | Reference |  |
|  | 18.5～24.9 | | 0.615 (0.403-0.940) |  | |  | 0.730 (0.469-1.137) |  |
|  | 25.0～29.9 | | 0.664 (0.441-1.002) |  | |  | 0.669 (0.435-1.030) |  |
|  | ≥30 | | 0.752 (0.501-1.127) |  | |  | 0.952 (0.619-1.465) |  |
| ASA score | | |  | 0.127 | |  |  |  |
|  | I or II | | Reference |  | |  |  |  |
|  | III or IV | | 1.263 (0.936-1.705) |  | |  |  |  |
| Tumor size, mm | | |  | 0.346 | |  |  |  |
|  | ＜40 | | Reference |  | |  |  |  |
|  | ＞40 | | 0.866 (0.642-1.169) |  | |  |  |  |
| History of abdominal surgery | | |  | 0.134 | |  |  |  |
|  | No | | Reference |  | |  |  |  |
|  | Yes | | 1.258 (0.932-1.699) |  | |  |  |  |
| History of smoking | | |  | 0.944 | |  |  |  |
|  | No | | Reference |  | |  |  |  |
|  | Yes | | 0.987 (0.697-1.399) |  | |  |  |  |
| History of drinking | | |  | 0.135 | |  |  |  |
|  | No | | Reference |  | |  |  |  |
|  | Yes | | 1.328 (0.916-1.925) |  | |  |  |  |
| Tumor location | | |  | 0.976 | |  |  |  |
|  | Colon | | Reference |  | |  |  |  |
|  | Rectum | | 0.995 (0.731-1.355) |  | |  |  |  |
| Medical Center | | |  | 0.158 | |  |  |  |
|  | | Xi'an cohort | Reference |  | |  |  |  |
|  | | Yulin cohort | 0.740 (0.488-1.124) |  | |  |  |  |
| Differentiation grade | | |  | 0.008 | |  |  | 0.007 |
|  | Well or moderate | | Reference |  | |  | Reference |  |
|  | Poor or worse | | 1.598 (1.128-2.263) |  | |  | 1.649 (1.146-2.373) |  |
| Surgical approach | | |  | 0.593 | |  |  |  |
|  | Minimally invasive surgery* | | Reference |  | |  |  |  |
|  | Open surgery | | 1.125 (0.731-1.732) |  | |  |  |  |
| Pathological T stage | | |  | ＜0.001 | |  |  | ＜0.001 |
|  | T1 | | Reference |  | |  | Reference |  |
|  | T2 | | 0.717 (0.346-1.485) |  | |  | 0.905 (0.430-1.983) |  |
|  | T3 | | 1.678 (0.956-2.946) |  | |  | 1.476 (0.817-2.667) |  |
|  | T4 | | 4.218 (2.335-7.621) |  | |  | 3.091 (1.619-5.902) |  |
| Pathological N stage | | |  | ＜0.001 | |  |  | 0.721 |
|  | N0 | | Reference |  | |  | Reference |  |
|  | N1 | | 1.285 (0.880-1.876) |  | |  | 0.905 (0.602-1.362) |  |
|  | N2 | | 2.654 (1.855-3.797) |  | |  | 1.093 (0.710-1.683) |  |
| Pathological M stage | | |  | ＜0.001 | |  |  | ＜0.001 |
|  | M0 | | Reference |  | |  | Reference |  |
|  | M1 | | 5.103 (3.617-7.199) |  | |  | 3.456 (2.342-5.099) |  |
| Neoadjuvant therapy | | |  | 0.188 | |  |  |  |
|  | Yes | | Reference |  | |  |  |  |
|  | No | | 1.274 (0.889-1.827) |  | |  |  |  |
| Adjuvant therapy | | |  | 0.031 | |  |  | 0.033 |
|  | Yes | | Reference |  | |  | Reference |  |
|  | No | | 2.451 (1.085-5.535) |  | |  | 2.459 (1.075-5.682) |  |
| CFS score | | |  | ＜0.001 | |  |  | 0.387 |
|  | Fit | | Reference |  | |  | Reference |  |
|  | Pre-frailty | | 0.553 (0.347-0.882) |  | |  | 0.734 (0.438-1.230) |  |
|  | Frailty | | 1.223 (0.765-1.955) |  | |  | 0.914 (0.521-1.603) |  |
| FP score | | |  | ＜0.001 | |  |  | 0.010 |
|  | Fit | | Reference |  | |  | Reference |  |
|  | Pre-frailty | | 3.942 (2.055-7.564) |  | |  | 3.036 (1.475-6.248) |  |
|  | Frailty | | 6.614 (3.401-12.863) |  | |  | 2.334 (1.282-5.429) |  |
| G-8 score | | |  | ＜0.001 | |  |  | 0.230 |
|  | Fit | | Reference |  | |  | Reference |  |
|  | Pre-frailty | | 0.684 (0.444-1.052) |  | |  | 0.633 (0.372-1.077) |  |
|  | Frailty | | 1.895 (1.329-2.702) |  | |  | 0.816 (0.532-1.252) |  |
| FI score | | |  | ＜0.001 | |  |  | 0.005 |
|  | Fit | | Reference |  | |  | Reference |  |
|  | Pre-frailty | | 1.783 (1.064-2.986) |  | |  | 1.242 (0.651-2.371) |  |
|  | Frailty | | 4.474 (2.828-7.081) |  | |  | 2.590 (1.162-5.773) |  |
| HR, hazard ratio; ASA, American Society of Anesthesiologists physical status classification; *, laparoscopic surgery or robotic surgery; CFS, Clinical Frailty Scale; FP, frailty phenotype; G-8, Geriatric-8; FI, frailty index. | | | | | | | | |

**Supplementary Table 14.** Univariate and multivariate analysis of risk factors for postoperative clinically relevant complications in older patients with colorectal cancer

| **Variables** | | **Univariate analysis** | |  | **Multivariate analysis^*^** | |
| --- | --- | --- | --- | --- | --- | --- |
|  |  | **Odds ratio (95% CI)** | ***p*-value** |  | **Odds ratio (95% CI)** | ***p*-value** |
| Age | |  | ＜0.001 |  |  | ＜0.001 |
|  | 65-69 | Reference |  |  | Reference |  |
|  | 70-74 | 1.509 (0.950-2.398) |  |  | 1.328 (0.794-2.221) |  |
|  | 75-79 | 1.818 (1.105-2.992) |  |  | 1.835 (1.056-3.188) |  |
|  | 80+ | 3.481 (2.009-6.031) |  |  | 3.444 (1.850-6.414) |  |
| Sex | |  | 0.485 |  |  |  |
|  | male | Reference |  |  |  |  |
|  | female | 0.882 (0.619-1.255) |  |  |  |  |
| BMI | |  | 0.237 |  |  | 0.082 |
|  | ＜18.5 | Reference |  |  | Reference |  |
|  | 18.5～24.9 | 0.950 (0.571-1.578) |  |  | 1.058 (0.589-1.903) |  |
|  | 25.0～29.9 | 0.789 (0.470-1.324) |  |  | 0.708 (0.391-1.283) |  |
|  | ≥30 | 1.313 (0.801-2.151) |  |  | 1.482 (0.836-2.628) |  |
| ASA score | |  | 0.323 |  |  | 0.239 |
|  | I or II | Reference |  |  | Reference |  |
|  | III or IV | 1.196 (0.838-1.707) |  |  | 0.782 (0.520-1.178) |  |
| Tumor size, mm | |  | 0.784 |  |  |  |
|  | ＜40 | Reference |  |  |  |  |
|  | ＞40 | 1.051 (0.738-1.497) |  |  |  |  |
| History of abdominal surgery | |  | 0.207 |  |  |  |
|  | No | Reference |  |  |  |  |
|  | Yes | 1.313 (0.860-2.003) |  |  |  |  |
| History of smoking | |  | 0.806 |  |  |  |
|  | No | Reference |  |  |  |  |
|  | Yes | 1.052 (0.702-1.577) |  |  |  |  |
| History of drinking | |  | 0.436 |  |  |  |
|  | No | Reference |  |  |  |  |
|  | Yes | 1.203 (0.755-1.918) |  |  |  |  |
| Tumor location | |  | 0.498 |  |  |  |
|  | Colon | Reference |  |  |  |  |
|  | Rectum | 1.133 (0.790-1.625) |  |  |  |  |
| Medical Center | |  | 0.185 |  |  |  |
|  | Xi'an cohort | Reference |  |  |  |  |
|  | Yulin cohort | 0.727 (0.454-1.165) |  |  |  |  |
| Differentiation grade | |  | 0.369 |  |  |  |
|  | Well or moderate | Reference |  |  |  |  |
|  | Poor or worse | 1.227 (0.786-1.915) |  |  |  |  |
| Surgical approach | |  | ＜0.001 |  |  | ＜0.001 |
|  | Minimally invasive surgery* | Reference |  |  | Reference |  |
|  | Open surgery | 4.880 (2.984-7.982) |  |  | 4.245 (2.478-7.272) |  |
| Clinical T stage | |  | 0.048 |  |  | 0.016 |
|  | T1-2 | Reference |  |  | Reference |  |
|  | T3-4 | 1.472 (1.004-2.160) |  |  | 1.716 (1.107-2.658) |  |
| Clinical N stage | |  | 0.974 |  |  | 0.993 |
|  | N0 | Reference |  |  | Reference |  |
|  | N1-2 | 1.006 (0.700-1.445) |  |  | 0.998 (0.661-1.508) |  |
| Neoadjuvant therapy | |  | 0.149 |  |  |  |
|  | No | Reference |  |  |  |  |
|  | Yes | 1.567 (0.852-2.884) |  |  |  |  |
| CFS score | |  | ＜0.001 |  |  | 0.885 |
|  | Fit | Reference |  |  | Reference |  |
|  | Pre-frailty | 1.260 (0.658-2.412) |  |  | 1.179 (0.554-2.512) |  |
|  | Frailty | 3.300 (1.677-6.493) |  |  | 1.230 (0.532-2..844) |  |
| FP score | |  | ＜0.001 |  |  | 0.012 |
|  | Fit | Reference |  |  | Reference |  |
|  | Pre-frailty | 1.703 (1.004-2.888) |  |  | 0.953 (0.505-1.798) |  |
|  | Frailty | 4.336 (2.452-7.668) |  |  | 2.320 (1.244-4.328) |  |
| G-8 score | |  | ＜0.001 |  |  | 0.820 |
|  | Fit | Reference |  |  | Reference |  |
|  | Pre-frailty | 1.352 (0.816-2.240) |  |  | 0.848 (0.400-1.798) |  |
|  | Frailty | 4.597 (2.865-7.376) |  |  | 1.005 (0.426-2.368) |  |
| FI score | |  | ＜0.001 |  |  | 0.005 |
|  | Fit | Reference |  |  | Reference |  |
|  | Pre-frailty | 2.753 (1.601-4.735) |  |  | 3.008 (1.314-6.887) |  |
|  | Frailty | 5.237 (3.121-8.787) |  |  | 3.130 (1.395-7.023) |  |
| **^*^** adjusted by age, BMI, ASA score, and TNM stage**;** ASA, American Society of Anesthesiologists physical status classification; *, laparoscopic surgery or robotic surgery; CFS, Clinical Frailty Scale; FP, frailty phenotype; G-8, Geriatric-8; FI, frailty index. | | | | | | |

**Supplementary Table 15.** Univariate and multivariate analysis of risk factors for one-year mortality in older patients with colorectal cancer

| **Variables** | | | **Univariate analysis** | | |  | **Multivariate analysis^*^** | |
| --- | --- | --- | --- | --- | --- | --- | --- | --- |
|  |  |  | **HR (95% CI)** | | ***p*-value** |  | **HR (95% CI)** | ***p*-value** |
| Age | | |  | 0.006 | |  |  | 0.002 |
|  | 65-69 | | Reference |  | |  | Reference |  |
|  | 70-74 | | 1.502 (0.865-2.608) |  | |  | 1.444 (0.810-2.575) |  |
|  | 75-79 | | 2.440 (1.417-4.201) |  | |  | 1.857 (1.033-3.338) |  |
|  | 80+ | | 2.329 (1.257-4.317) |  | |  | 3.661 (1.841-7.279) |  |
| Sex | | |  | 0.850 | |  |  |  |
|  | male | | Reference |  | |  |  |  |
|  | female | | 1.039 (0.700-1.542) |  | |  |  |  |
| BMI | | |  | 0.319 | |  |  | 0.381 |
|  | ＜18.5 | | Reference |  | |  | Reference |  |
|  | 18.5～24.9 | | 0.614 (0.351-1.072) |  | |  | 0.672 (0.375-1.204) |  |
|  | 25.0～29.9 | | 0.676 (0.393-1.164) |  | |  | 0.768 (0.440-1.340) |  |
|  | ≥30 | | 0.765 (0.450-1.301) |  | |  | 1.055 (0.605-1.840) |  |
| ASA score | | |  | 0.513 | |  |  | 0.879 |
|  | I or II | | Reference |  | |  | Reference |  |
|  | III or IV | | 1.141 (0.768-1.695) |  | |  | 1.032 (0.687-1.551) |  |
| Tumor size, mm | | |  | 0.607 | |  |  |  |
|  | ＜40 | | Reference |  | |  |  |  |
|  | ＞40 | | 0.902 (0.608-1.337) |  | |  |  |  |
| History of abdominal surgery | | |  | 0.257 | |  |  |  |
|  | No | | Reference |  | |  |  |  |
|  | Yes | | 1.257 (0.847-1.864) |  | |  |  |  |
| History of smoking | | |  | 0.984 | |  |  |  |
|  | No | | Reference |  | |  |  |  |
|  | Yes | | 1.005 (0.639-1.581) |  | |  |  |  |
| History of drinking | | |  | 0.380 | |  |  |  |
|  | No | | Reference |  | |  |  |  |
|  | Yes | | 1.251 (0.759-2.063) |  | |  |  |  |
| Tumor location | | |  | 0.909 | |  |  |  |
|  | Colon | | Reference |  | |  |  |  |
|  | Rectum | | 1.024 (0.683-1.535) |  | |  |  |  |
| Medical Center | | |  | 0.296 | |  |  |  |
|  | | Xi'an cohort | Reference |  | |  |  |  |
|  | | Yulin cohort | 0.742 (0.428-1.285) |  | |  |  |  |
| Differentiation grade | | |  | 0.444 | |  |  |  |
|  | Well or moderate | | Reference |  | |  |  |  |
|  | Poor or worse | | 1.207 (0.746-1.954) |  | |  |  |  |
| Surgical approach | | |  | 0.659 | |  |  |  |
|  | Minimally invasive surgery* | | Reference |  | |  |  |  |
|  | Open surgery | | 1.136 (0.645-1.999) |  | |  |  |  |
| Pathological T stage | | |  | ＜0.001 | |  |  | 0.013 |
|  | T1 | | Reference |  | |  | Reference |  |
|  | T2 | | 0.733 (0.283-1.990) |  | |  | 0.691 (0.261-1.828) |  |
|  | T3 | | 1.647 (0.784-3.460) |  | |  | 1.040 (0.478-2.264) |  |
|  | T4 | | 3.995 (1.802-8.680) |  | |  | 2.100 (0.895-4.926) |  |
| Pathological N stage | | |  | ＜0.001 | |  |  | 0.496 |
|  | N0 | | Reference |  | |  | Reference |  |
|  | N1 | | 1.329 (0.810-2.181) |  | |  | 0.976 (0.577-1.652) |  |
|  | N2 | | 2.732 (1.708-4.372) |  | |  | 1.344 (0.771-2.341) |  |
| Pathological M stage | | |  | ＜0.001 | |  |  | ＜0.001 |
|  | M0 | | Reference |  | |  | Reference |  |
|  | M1 | | 4.691 (2.977-7.393) |  | |  | 3.206 (1.949-5.273) |  |
| Neoadjuvant therapy | | |  | 0.232 | |  |  |  |
|  | Yes | | Reference |  | |  |  |  |
|  | No | | 1.883 (0.597-5.942) |  | |  |  |  |
| Adjuvant therapy | | |  | 0.207 | |  |  |  |
|  | Yes | | Reference |  | |  |  |  |
|  | No | | 1.364 (0.842-2.208) |  | |  |  |  |
| CFS score | | |  | 0.003 | |  |  | 0.692 |
|  | Fit | | Reference |  | |  | Reference |  |
|  | Pre-frailty | | 0.611 (0.330-1.130) |  | |  | 0.800 (0.397-1.609) |  |
|  | Frailty | | 1.266 (0.677-2.367) |  | |  | 0.725 (0.346-1.518) |  |
| FP score | | |  | ＜0.001 | |  |  | 0.231 |
|  | Fit | | Reference |  | |  | Reference |  |
|  | Pre-frailty | | 3.702 (1.594-8.599) |  | |  | 2.256 (0.885-5.751) |  |
|  | Frailty | | 6.151 (2.600-14.552) |  | |  | 2.256 (0.790-6.448) |  |
| G-8 score | | |  | ＜0.001 | |  |  | 0.562 |
|  | Fit | | Reference |  | |  | Reference |  |
|  | Pre-frailty | | 0.737 (0.417-1.300) |  | |  | 0.700 (0.319-1.534) |  |
|  | Frailty | | 1.974 (1.231-3.167) |  | |  | 1.063 (0.570-1.982) |  |
| FI score | | |  | ＜0.001 | |  |  | 0.016 |
|  | Fit | | Reference |  | |  | Reference |  |
|  | Pre-frailty | | 1.715 (0.873-3.368) |  | |  | 1.272 (0.525-3.085) |  |
|  | Frailty | | 4.297 (2.363-7.814) |  | |  | 2.803 (1.278-6.294) |  |
| **^*^** adjusted by age, BMI, ASA score, and TNM stage**;** HR, hazard ratio; ASA, American Society of Anesthesiologists physical status classification; *, laparoscopic surgery or robotic surgery; CFS, Clinical Frailty Scale; FP, frailty phenotype; G-8, Geriatric-8; FI, frailty index. | | | | | | | | |
